# Digital Mapping of Soil Salinity and Crop Yield across a Coastal Agricultural Landscape Using Repeated Electromagnetic Induction (EMI) Surveys

**DOI:** 10.1371/journal.pone.0153377

**Published:** 2016-05-20

**Authors:** Rongjiang Yao, Jingsong Yang, Danhua Wu, Wenping Xie, Peng Gao, Wenhui Jin

**Affiliations:** 1 State Key Laboratory of Soil and Sustainable Agriculture, Institute of Soil Science, Chinese Academy of Sciences, Nanjing, 210008, China; 2 Dongtai Institute of Tidal Flat Research, Nanjing Branch of the Chinese Academy of Sciences, Dongtai, 224200, China; 3 Department of Geography, University of South Carolina, 709 Bull Street, Columbia, South Carolina, 29208, United States of America; Estación Experimental del Zaidín (CSIC), SPAIN

## Abstract

Reliable and real-time information on soil and crop properties is important for the development of management practices in accordance with the requirements of a specific soil and crop within individual field units. This is particularly the case in salt-affected agricultural landscape where managing the spatial variability of soil salinity is essential to minimize salinization and maximize crop output. The primary objectives were to use linear mixed-effects model for soil salinity and crop yield calibration with horizontal and vertical electromagnetic induction (EMI) measurements as ancillary data, to characterize the spatial distribution of soil salinity and crop yield and to verify the accuracy of spatial estimation. Horizontal and vertical EMI (type EM38) measurements at 252 locations were made during each survey, and root zone soil samples and crop samples at 64 sampling sites were collected. This work was periodically conducted on eight dates from June 2012 to May 2013 in a coastal salt-affected mud farmland. Multiple linear regression (MLR) and restricted maximum likelihood (REML) were applied to calibrate root zone soil salinity (EC_e_) and crop annual output (CAO) using ancillary data, and spatial distribution of soil EC_e_ and CAO was generated using digital soil mapping (DSM) and the precision of spatial estimation was examined using the collected meteorological and groundwater data. Results indicated that a reduced model with EM_h_ as a predictor was satisfactory for root zone EC_e_ calibration, whereas a full model with both EM_h_ and EM_v_ as predictors met the requirement of CAO calibration. The obtained distribution maps of EC_e_ showed consistency with those of EMI measurements at the corresponding time, and the spatial distribution of CAO generated from ancillary data showed agreement with that derived from raw crop data. Statistics of jackknifing procedure confirmed that the spatial estimation of EC_e_ and CAO exhibited reliability and high accuracy. A general increasing trend of EC_e_ was observed and moderately saline and very saline soils were predominant during the survey period. The temporal dynamics of root zone EC_e_ coincided with those of daily rainfall, water table and groundwater data. Long-range EMI surveys and data collection are needed to capture the spatial and temporal variability of soil and crop parameters. Such results allowed us to conclude that, cost-effective and efficient EMI surveys, as one part of multi-source data for DSM, could be successfully used to characterize the spatial variability of soil salinity, to monitor the spatial and temporal dynamics of soil salinity, and to spatially estimate potential crop yield.

## Introduction

Soil salinization in the coastal zone of the Yangtze River alluvial sediments in Eastern China is a constant threat to agriculture and ecology. Among them, the coastal region of Jiangsu Province has possessed a total of about 8 ×10^5^ ha salinized soil resources including mud flats, accounting for over one quarter of total tidal flats in China [1]. These soils are naturally saline due to marine immersion, the presence of a shallow, saline water table and coarse soil texture. Although this area has been experiencing a slowly reduce of soil salinity owing to substantial rainfall [2], farmers annually suffer from over 30% yield reduction due to large evaporation/precipitation (E/P) ratio in dry season, low leaching efficiency in rain season as well as lack of reliable soil salinity monitoring. Accurate and real-time salinity information becomes increasingly important for developing management strategies that aim to minimize salinization and maximize crop output in this area.

Rapid and reliable methods for obtaining information on the field soil salinity have made great progress in the last two decades. Recently, rapid techniques of remote sensing and proximal sensory, which provided favorable facilities for detecting soil salinity and other properties, have attracted more interests [3–5]. The most widely used technique is proximal sensing electromagnetic induction (EMI) instruments including the EM31, EM38, EM38-DD, and EM38-MK2 meters, the DUALEM-1 and DUALEM-2 meters, and the Profiler EMP-400 [6]. These EMI sensors gauge the apparent soil electrical conductivity (EC_a_) with the advantages such as high speed, ease of use, relatively low cost, and large volume of data collected over traditional methods [7]. Up to the present, EMI sensors have found wide applications in fields of precision agriculture, water-saving irrigation, hydrological and pedological processes. The success lies in the fact that EMI readings are easily correlated to soil attributes in the rootzone [8], vadose zone [9] and deeper regolity [10], and these correlations have then been used to map soil attributes from field to landscape scales [11–12]. However, the response of apparent electrical conductivity measured by EMI techniques to soil salinity is influenced by a wide range of indirect factors, such as soil moisture, clay content, bulk density and mineralogy [13–14]. The real challenge is that EMI techniques work best in areas where there are large changes in one soil property that influences soil electrical conductivity, and do not work as well when soil properties that influence electrical conductivity are largely homogenous [15].

The primary use of proximal sensing EMI instruments in agriculture is for the assessment of soil salinity at different scales and EM38 meter has been the most widely used EMI sensor in soil science [16]. This meter provides an effective exploration depth of 0.75 and 1.5 m when it is operated in the horizontal and vertical dipoles, respectively. Using the EM38 meter measurements as ancillary data, the spatial variability of EMI data has been widely used to better infer the spatial variability of soils salinity, water content, clay content, cation exchange capacity, and even soil depth [17–20]. More recently, EM38 meter has gained popularity in precision farming, such as improvement of soil mapping [21], potential crop yield estimation with the combination of satellite imagery [22], identification of manure accumulation area and soil constraints to the crop yields [23], and assessment of potential nutrient build-up [24]. In addition, EM38 meter has been attracting attentions of researchers who are interested in precise delineation of field soil salinity, and appraisal and modeling of related agricultural managements on crop growth, salt transport and water usage [25–27].

Despite the successful application of EMI in many regions around the world, few studies have examined the use of periodical EMI survey measurements as ancillary data to estimate the field soil salinity and crop yield in the marine-terrestrial interlaced region of the coastal zone of Jiangsu Province. In this study, repeated EMI surveys with EM38 meter and digital soil mapping (DSM) were employed to characterize the spatial distribution and temporal dynamics of root zone soil salinity on eight survey dates, and to map the spatial pattern of crop annual output (CAO) based upon ancillary data. This work was conducted in a salt-affected agricultural landscape which was enclosed and reclaimed from coastal mudflats in 2004. The primary objectives were: (*i*) to establish the relationship between root zone salinity, CAO and the EMI measurements using multiple linear regression (MLR) and restricted maximum likelihood (REML), (*ii*) to evaluate the spatial distribution and temporal changes of soil salinity on different survey dates, and to investigate the reliability of the estimation of spatial soil salinity using weather and groundwater data, and (*iii*) to spatially estimate CAO using the average EMI measurements obtained from the eight surveys as ancillary data and to verify the feasibility and precision of CAO prediction procedure.

## Materials and Methods

### Ethics Statement

We selected a coastal salt-affected rainfed field in the Huanghai Raw Seed Growing Farm, located in the southeast region of Dongtai Prefecture, Jiangsu Province, China to conduct this study. One field, which was approximately 0.93 ha and reclaimed from coastal mud flats in 2004, was used to perform soil sampling and repeated electromagnetic induction (EMI) surveys from June 2012 to May 2014. This study was permitted by the Agricultural Commission of Dongtai Prefecture. No endangered or protected species were involved in the study.

### Experimental site characteristics

**Fig 1:**
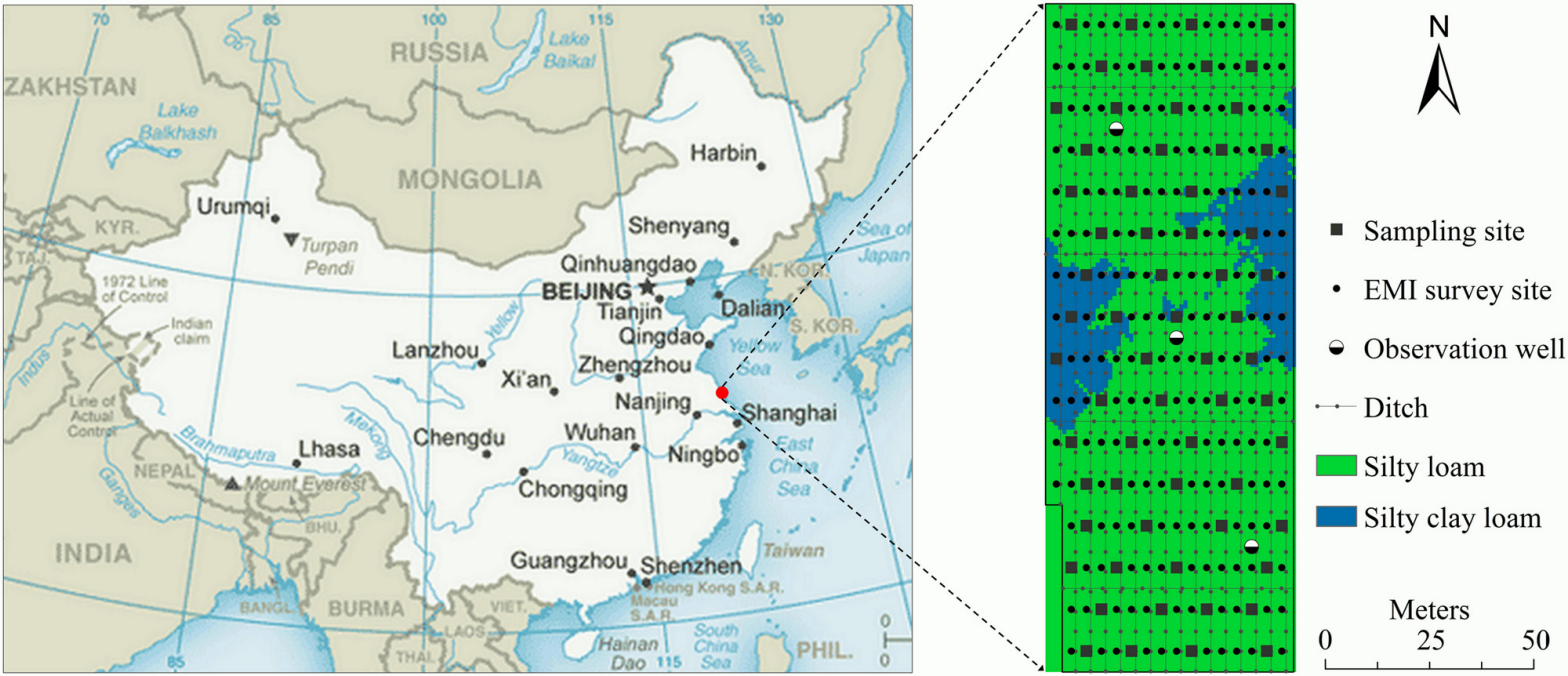
Geographical location of the experimental site and spatial distribution of soil texture, field infrastructures, soil sampling sites and EMI survey sites. Data source of the map of China in the left graph: Central Intelligence Agency (https://www.cia.gov/library/publications/the-world-factbook/index.html, last access March 21, 2016).

The experiment site was the Huanghai Raw Seed Growing Farm with central coordinates 32°39′N and 120°53′E, situated in the marine-terrestrial interlaced area, southeast of Dongtai Prefecture, Jiangsu Province, China (Fig 1). The site has a typical coastal salt-affected agricultural landscape in the subtropical zone of East China, and is characterized by the southeast monsoon from spring to autumn and the northwest monsoon in winter owing to the oceanic and continental climate. The distance from the site to the Yellow Sea Coastline is approximately seven kilometers and this farm has nearly flat topography, with an elevation of 1.0–1.5 m above sea level. The land of this farm, enclosed and reclaimed from coastal mudflats in 1999 and 2004, respectively, is divided by dikes of different ages running in a north-south direction (see [28]). The predominant soil is silt loam in texture, developed from the alluvial sediments of the Yangtze River and Huai River and marine sediments [29], and is classified as a loamy, mixed, hyperthermic, Aquic Halaquepts according to USDA soil taxonomy [30].

### Land use and management history

A section of land between Dike1999 and Dike2004, which was approximately 0.93 ha and reclaimed from coastal mud flats in 2004, was chosen as the experimental site (Fig 1). The experimental site had no documented history of cultivation prior to April, 2006. A rice/ barley rotation, which is a widely used rotation system in coastal salt-affected farmlands, has been practiced in the experimental site. The rice (*Oryza sativa* L.) variety was Huaidao 9 (a japonica inbred) and the barley (*Hordeum vulgare* L.) variety was Supi 4. Rice paddies were initially constructed to leach soil salinity because the salt levels of the newly-reclaimed land exceeded the salt tolerant thresholds for most agricultural crops. Fresh water used for paddy rice was pumped from the underground wells at approximately 300–400 m depth with an EC (electrical conductivity) of 0.47 dS m^-1^. Due to the continuous decline of the water table in this area, the amount of fresh water from these wells no longer met the water demand for rice production on all fields. Therefore, rainfed corn/ barley rotation has been increasingly chosen by farmers and Suyu 20 was the most widely used corn hybrid variety (*Zea mays* L.) in this farm.

On the experimental site, rainfed rotation began from the barley season of 2009, and conventional soil fertility and pest management practices have been uniformly used. Using diammonium phosphate as basal fertilizer, a total of 450 kg/ha N and 180 kg/ha P_2_O_5_ has been applied in corn and barley seasons and no potassium fertilizer has been used. Crop residues were the main source of organic matter inputs. High soil salinity, coarse soil texture and poor soil nutrient supplying capacity are known as the most significant limitations to soil productivity and crop growth varies greatly in the experimental site due to spatial variation of soil conditions.

### Field EMI survey

Repeated EMI (electromagnetic induction) surveys were conducted on the experimental site. During each survey, uniform grids with an interval of 3.6 m from west to east and 10 m from north to south were imposed on EMI measurement sites, and a total of 252 EMI measurement sites consisting of 16 east-west direction transects were determined across the experimental site (Fig 1). At each site, an electromagnetic induction instrument (type EM38) in the horizontal and vertical operation dipoles was positioned on the soil surface, and the measurements were taken and recorded (denoted by EM_h_ and EM_v_), respectively. The intensive EMI survey was conducted on 8 dates between June 2012 and May 2013, with each EMI survey completed in two consecutive days (S1 File). The interval between 8 surveys ranged from 1 to 3 months. During each EMI survey, the soil temperature of the experimental site at 2, 5, 10, 20 and 40 cm layers was hourly monitored using an electronic thermometer with a metal probe. To avoid a proportional shift due to differences in temperature, the recorded EMI measurements were manually calibrated to values at 25°C according to Ma et al. [31]. EMI surveys were not performed in rainy days to prevent the adverse impact of rainwater on the instrument, and no precipitation occurred during the 8 survey dates (S2 File). Thus, the variation of soil water content during each survey time was considered negligible. The influences of soil texture, terrain and bulk density on EMI measurements were also neglected considering the flat topography and uniform management practices in the experimental site. Table 1 shows the linear regression parameters between the horizontal and vertical EMI measurements obtained on the 8 survey dates across the experimental site. A strong linear relationship was observed between the measured EM_h_ and EM_v_ data, indicating the reliability of apparent electrical conductivity (EC_a_) data during each EMI survey.

**Table 1:** Regression parameters between the horizontal and vertical EMI measurements on different survey dates (*n* = 252).

			EM_h_ = a+b·EMv			
Survey date	Elapsed time	Weather conditions	*a*	*b*	R^2^	RMSE
8 June 2012	8–9 June 2012	Cloudy	-13.40	0.84	0.92	24.18
31 July 2012	31 July–1 August 2012	Cloudy	-18.29	0.97	0.91	32.64
3 September 2012^†^	3–4 September 2012	Sunny	-24.52	1.06	0.89	46.81
11 December 2012	11–12 December 2012	Sunny	-22.20	0.96	0.90	47.28
12 January 2013	12–13 January 2013	Sunny	-24.33	1.00	0.89	54.55
24 February 2013	24–25 February 2013	Sunny	16.27	0.85	0.73	75.41
31 March 2013^†^	31 March–1 April 2013	Cloudy	-31.70	1.08	0.99	49.43
23 May 2013	23–24 May 2013	Sunny	-15.79	1.09	0.90	40.46

EM_h_, EMI measurements made in the horizontal operation dipole, mS/m; EM_v_, EMI measurements made in the vertical operation dipole, mS/m;

^†^ indicates the date of soil sampling; the same below

### Soil sampling and lab analyses

Among 16 east-west direction EMI survey transects, 64 locations were randomly selected for soil sampling with four locations in each transect. Just beneath the EMI measurement position, soil samples were collected by hand augering at 0–0.2, 0.2–0.4, 0.4–0.6, 0.6–0.8 and 0.8–1.0 m layers for laboratory analyses of soil salinity. At each location, soil sample of each layer was determined using a quartering method, and this work was carried out simultaneously with field EMI survey. Soil sampling was conducted only on two survey dates (i.e., 3 September 2012 and 31 March 2013). Therefore, soil samples of a total of 128 cores were obtained for the calibration of EMI measurements (S1 File).

All soil samples were air-dried and passed through a 2 mm sieve prior to lab analyses. Soil salinity was determined using EC_1:5_ (electrical conductivity of 1:5 soil/water paste extract). In addition, soil samples of 32 cores were randomly selected for the analysis of EC_e_ (electrical conductivity of saturated soil paste extract) on each soil sampling date according to the procedure by the U.S. Salinity Laboratory Staff [32]. A strong positive linear relationship was observed between EC measurements of the two methods. The regression equation relating EC_e_ (dS/m) to EC_1:5_ (dS/m) was given by:

$$EC_{e}=9.127EC_{1:5}+0.635\;n=160$$ (1)

This relationship (*r*^2^ = 0.943) was then employed to convert EC_1:5_ to EC_e_ for the soil samples which EC_e_ was not measured. This equation shows agreement with the relationships reported by Slavich and Petterson [33] for silt loam soil and by Yao et al. [34] in the similar region. In this study, root zone EC_e_ (i.e., the average EC_e_ value of 0–1.0 m soil solum) was determined and used for mapping soil salinity at different time stamps.

### Meteorological and groundwater data collection

Table 2 shows the monthly meteorological and groundwater data during the whole survey period. The meteorological data was collected from the weather station located in the experimental site and the groundwater data, including water table and groundwater salinity was recorded with CTD-Divers (type DI263) installed in the observation wells at the experimental site (Table 2). The meteorological and groundwater data were hourly collected.

**Table 2:** Monthly meteorological and groundwater data from 1 June 2012 to 31 May 2013 in our experimental site.

	Months												
Weather and groundwater parameters	Jun.	Jul.	Aug.	Sept.	Oct.	Nov.	Dec.	Jan.	Feb.	Mar.	Apr.	May	Total/average
Temperature (°C)	24.1	27.3	26.7	23.4	17.7	11.1	4.1	1.8	4.4	8.3	14.1	19.4	15.2
Relative humidity (%)	69.2	86.1	80.4	78.5	70.2	74.6	75.8	71.5	77.4	73.6	79.2	77.2	76.1
Wind speed (m/s)	2.9	2.8	2.4	2.2	2.4	2.3	3.0	2.6	3.2	3.5	3.1	3.2	2.8
Insolation duration (h)	155.2	158.2	192.6	186.1	172.4	165.2	147.2	146.4	132.7	177.8	187.4	214.5	2035.7
Precipitation (mm)	12.7	339.6	167.0	80.7	30.2	61.6	57.2	16.0	55.6	18.1	56.6	51.8	947.1
Evaporation (mm)	104.4	126.2	108.2	96.7	88.3	64.5	43.6	38.1	45.3	73.8	102.7	130.0	1021.8
Water table (m)	2.32	0.95	1.19	0.87	2.16	1.36	0.87	1.00	0.57	1.72	2.37	2.61	1.5
Groundwater salinity (dS/m)	7.28	27.46	28.69	28.98	10.78	28.22	29.54	30.98	30.56	16.79	10.07	11.99	21.8

Out of the total rainfall of 947.1 mm, the amount during the corn growing season (from sowing time in June 2012 to harvesting time in October 2012) was 630.2 mm accounting for 66.5%, whereas that during the barley growing season (from sowing time in November 2012 to harvesting time in May 2013) was 316.9 mm occupying 33.5%. Monthly mean air temperature varied from 1.8°C in January to 27.3°C in July with an annual average of 15.2°C. Monthly mean evaporation, measured with the E-601 type of evaporimeter, ranged from 38.1 mm in January to 130.0 mm in May with an annual value of 1021.8 mm. The annual insolation duration was 2035.7 hours and the experimental site got 76.1% of average relative humidity and 2.8 m/s of average wind velocity. The monthly mean water table fluctuated between 0.57 m in February 2013 and 2.61 m in May 2013 with an annual average of 1.5 m. The monthly mean groundwater salinity varied from 7.28 dS/m in June 2012 to 30.98 dS/m in January 2013 with an annual average of 21.8 dS/m.

### Crop yield determination

At each soil sampling location, crop yields of corn and barley were measured at their harvest seasons. At the end of corn season, corn cobs of 80 plants were picked manually from eight rows of ten plants each, and 20 plants in adjacent two rows representing a replica were also collected. Area of each replica was 4 m^2^ as the corn population density was five plants m^-2^ with average row spacing of 0.80 m and plant spacing of 0.25 m. This work was done in the middle of October 2012. At the end of the barley season, four replicas of above-ground barley spaced 5 m apart were manually cut from the four 1 m × 1 m plots at each location. This was carried out in late May 2013. Grains of corn and barley were threshed using a miniature thresher for each replica, and yield of corn and barley was determined by weighing the grains after oven drying at 60°C. Considering the uniform management practices and fertilizer application in the experiment site, crop annual output (CAO), which was the sum of corn yield and barley yield in one corn/barley rotation, was used as the soil productivity in this study (S1 File).

### Linear mixed-effects model (LME)

Soil salinity and crop yield were predicted using EMI measurements obtained at horizontal and vertical operation dipoles as ancillary data. For soil salinity estimation, EMI measurements made on 3 September 2012 and 31 March 2013 were used as ancillary variables of soil EC_e_, whereas the average EMI measurements (EM_ave_) during our investigation periods were used as ancillary variables for CAO estimation. Considering the data used and expected errors were spatially autocorrelated, a linear mixed-effects model (LME), which allows to model a spatially correlated outcome [35], was employed to fit the relationship between EC_e_ and EMI measurements (i.e., EM_h_ and EM_v_) as well as CAO and the average EMI measurements (i.e., EM_h_ave_ and EM_v_ave_). Linear mixed effects models simply model the fixed and random effects as having a linear form. Using the familiar notation, the linear mixed effect model takes the form:

y=Xβ+η+ε (2)

where *y* is a *n*×1 vector of values of the target variable, *X* is a *n*×*p* data matrix, *β* is a *p*×1 vector of fixed-effect regression coefficients, *η* is a *n*×1 vector, the elements of which are a realization of a spatially autocorrelated random variable, and *ε* is a *n*×1 vector, the elements of which are a realization of an independent and identically distributed random variable. There is one element equal to 1 in each row of the data matrix. Thus, the elements of *β* are the estimated mean values of the target soil variable in the corresponding classes. The autocorrelated random variable *η* is assumed to be normal with mean zero and variance parameters. The error variable *ε* also has zero mean and a variance *σ*_*ε*_^2^.

The model in Eq 2 was fitted for the target variables (i.e., root zone EC_e_ and crop annual output) and with the fixed-effects of a subset of the ancillary variables in a regression type model. In the linear mixed model fitting procedure, variance parameters for the random effects are first estimated by restricted maximum likelihood (REML) and the fixed-effects coefficients are then estimated by weighted least squares. More details of the method used are described in Lark et al. [36].

### Restricted maximum likelihood (REML)

A full model with continuous fixed effects was initially selected to fit root zone EC_e_ (or CAO) with EM_h_ and EM_v_ (or EM_h_ave_ and EM_v_ave_) as predictors. A comparison was then made between this full model and a series of predictor-reduced models which were generated by leaving each predictor out in turn. In the comparison, the log-likelihood ratio of all models was computed and tested using chi-squared test with one degree of freedom [37]. The criterion of rejecting a predictor was that the reduced model developed by dropping this predictor was not significantly worse than the full model. This procedure was repeated until no further predictors were rejected. Since restricted likelihoods cannot be compared between models and different fixed effects, maximum likelihood was employed in this procedure and the model was re-estimated by REML after the predictors were determined.

### Statistical Analysis

Exploratory statistics of EMI survey measurements at different periods and the average EMI measurements was performed using the software SPSS [38], and the normality of the distribution was tested using one-sample Kolmogorov-Smirnov (K-S) test (*p* ≤ 0.05). Analysis of linear mixed-effects model (LME) and restricted maximum likelihood (REML) were also done in the software SPSS [39]. Spatial distribution of ancillary variables (i.e., proximally sensed EM38 at different periods) was first generated using ordinary kriging (OK). Spatial distribution of root zone EC_e_ and CAO was then generated from maps of ancillary variables using the reduced multiple linear regression (MLR) model and REML on a 1-m grid. The OK and spatial analysis procedure were carried out in ArcGIS 9.3 environment [40] and this work was performed for each EMI survey, respectively. Spatial accuracy of EC_e_ and CAO was assessed using jackknifing method on the soil sampling locations and two prediction criteria including the mean error (ME) and the root mean-square error (RMSE) were considered:

$$ME=\frac{1}{n}\overset{n}{\underset{i=1}{\sum }}[M(x_{i})-\text{P}(x_{i})]$$ (3)

$$RMSE=\sqrt{\frac{1}{n}\overset{n}{\underset{i=1}{\sum }}{\left[M(x_{i})-\text{P}(x_{i})\right]}^{2}}$$ (4)

Where *M*(*x*_*i*_) is the measured value at location *x*_*i*_, *P*(*x*_*i*_) is the predicted value at location *x*_*i*_, *n* is the number of locations in the jackknifing procedure. In this study, *n* equals to 128 for root zone EC_e_ as soil samples were collected on two survey dates. For CAO, *n* was set as 64 in that annual crop yield was used.

## Results and Discussion

### Exploratory data analysis

Table 3 shows the descriptive statistics of EMI measurements collected on different survey dates across the experimental site. Apparently, seasonal dynamics of apparent electrical conductivity was observed from the statistics, indicating the fluctuations of soil salinity over the study period. When the temporal change of EMI measurements was considered, the fifth survey date (i.e., 12 January 2013) had the highest mean EMI values ranging between 92.1 mS/m and 947.4 mS/m for EM_h_ and ranging from 65.8 mS/m to 870.3 mS/m for EM_v_. The lowest average EM_h_ and EM_v_ measurements occurred on 8 June 2012, indicating the lowest soil salinity at the first EMI survey time. Another indication was that soil salinity exhibited an increasing trend during our investigation period. It was also observed that EM_v_ data were larger than EM_h_ at most of the survey dates except 23 May 2013. In such an instance, normal distribution of soil salinity in the profile (i.e., increasing with depth) was suggested according to Corwin and Rhoades [41].

**Table 3:** Descriptive statistics of EM_h_ and EM_v_ measurements (mS/m) obtained on different survey dates.

	EM_h_/EM_ave_h_						EM_v_/EM_ave_v_					
Survey date	Min.	Max.	Mean	C_v_ (%)	Skew.	K-S p*	Min.	Max.	Mean	C_v_ (%)	Skew.	K-S p*
8 June 2012	14.8	593.9	173.5	49.0	1.8	0.25	55.9	574.9	221.4	43.6	1.0	0.43
31 July 2012	69.2	686.8	218.1	51.0	1.6	0.16	61.3	607.7	243.5	45.0	1.0	0.19
3 September 2012	57.9	858.4	277.7	51.4	1.4	0.51	68.7	697.4	283.8	44.6	0.9	0.13
11 December 2012	77.6	858.6	301.0	50.3	1.2	0.63	20.7	813.8	336.3	44.5	0.6	0.27
12 January 2013	92.1	947.4	327.6	51.4	1.4	0.47	65.8	870.3	353.0	45.3	0.8	0.51
24 February 2013	79.8	855.8	276.8	52.3	1.5	0.33	75.2	1090.5	305.5	47.4	1.3	0.13
31 March 2013	55.1	826.3	258.6	56.4	1.4	0.41	50.8	666.7	269.5	47.3	0.8	0.39
23 May 2013	59.0	711.8	256.0	49.4	1.1	0.32	57.9	607.0	251.1	44.1	0.7	0.49
EM_ave_	72.1	773.6	261.2	50.8	1.4	0.38	70.0	684.0	283.0	44.1	0.8	0.18

Min., minimum; Max., maximum; C_v_, coefficient of variation; Skew., skewness; K-S p*, significance level of Kolmogorov-Smirnov (K-S) normality test on log-transformed EMI measurements; EM_ave_, the average value of the eight EMI surveys; EM_ave_h_, the average horizontal EMI value of the eight surveys; EM_ave_v_, the average vertical EMI value of the eight surveys; the same below

For the most part, the various statistics derived from the eight surveys and from the average of the eight surveys were similar. This was the case for the coefficient of variation and skewness. The frequency distribution of EMI measurements was all characterized by left-skewed and low EMI value had higher frequency, which was witnessed by positive skewness ranging between 0.6 and 1.8. The EM_h_ measurements had larger extent of skewness than EM_v_ measurements. In fact, this left-skewed distribution indicates an evolving process of alleviation of soil salinization resulting from the agricultural utilization after the reclamation [34]. In order to satisfy the Gaussian assumption, normal transformation was deemed necessary for both EM_h_ and EM_v_. The results of one-sample Kolmogorov-Smirnov (K-S) normality test (*p*<0.05, two-tailed) showed that the EMI measurements were essentially normal distributed after logarithmic transformation (Table 3).

Table 4 shows the summary statistics of EC_e_ and CAO collected at 64 sampling locations. The average soil salinity EC_e_ on the two survey dates was 8.63 dS m^-1^ and 8.74 dS m^-1^, which exceeded the generally salt tolerance threshold for most agricultural crops [42]. The average crop annual output was 5.58 Mg/ha, classified as low soil productivity when compared with the high-and-middle yielding fields in the similar area due to high surface soil salinity and poor soil quality [43]. Strong correlation between EC_e_ at two survey times was observed (*r*^2^ = 0.92), indicating that the spatial and temporal similarity of soil salinity really existed due to uniform management practices used in the experimental site. Strong negative correlation was observed between CAO and EC_e_ on the two survey dates with correlation coefficient ranging between -0.46 and -0.49. It was not unexpected as excessively high soil salinity imposed significantly adverse impact on soil productivity in the coastal area.

**Table 4:** Descriptive statistics of EC_e_ (dS/m) and CAO (Mg/ha) collected at 64 sampling locations.

	EC_e_ (3 Sept. 2012)	EC_e_ (31 Mar. 2013)	CAO
Min.	1.56	1.40	0.81
Max.	28.42	28.77	11.24
Mean	8.63	8.74	5.58
Median	6.93	6.91	5.43
C_v_ (%)	60.21	65.52	36.66
Skew.	1.89	1.72	0.09
K-S p*	0.49	0.53	0.64
Pearson’s correlation coefficients squared			
EC_e_ (3 Sept. 2012)	1	0.92**	-0.46**
EC_e_ (31 Mar. 2013)		1	-0.49**
CAO			1

** Significant (two-tailed) at *p* < 0.01 by least significant difference (LSD)

### Log-likelihood analysis using REML

The correlation coefficients between EC_e_ and EMI measurements and between CAO and the average EMI measurements are shown in Table 5. To examine whether better correlations could be achieved between target variable and ancillary variables, we log-transformed EM_h_, EM_v_, EM_ave_h_ and EM_ave_v_ owing to that EMI data and the average EMI data were left skewed. Apparently, the correlation between EC_e_ and log-transformed EM_h_ and EM_v_ was not improved. This was also the case for CAO and log-transformed EM_ave_h_ and EM_ave_v_. Therefore, two reduced models which used EM_h_ and EM_v_ as ancillary variable, respectively, were determined for EC_e_ prediction, whereas EM_ave_h_ and EM_ave_v_ were employed as ancillary variable of the two reduced models for CAO prediction. Log-likelihood ratio test statistics of the selected reduced models are shown in Table 6.

**Table 5:** Correlation coefficients between EC_e_ and EMI measurements and between CAO and the average EMI measurements.

	EC_e_ (*n* = 128)	CAO (*n* = 64)
EM_h_	0.98**	
EM_v_	0.82**	
Ln-EM_h_	0.85**	
Ln-EM_v_	0.63**	
EM_ave_h_		-0.45**
EM_ave_v_		-0.37**
Ln-EM_ave_h_		-0.42**
Ln-EM_ave_v_		-0.29**

Significant (two-tailed) at *p* < 0.05 by least significant difference (LSD);

** Significant (two-tailed) at *p* < 0.01 by least significant difference (LSD)

**Table 6:** Log-likelihood ratio of reduced models for predicting EC_e_ and CAO.

	EM_h_/EM_ave_h_	EM_v_/EM_ave_v_	Selected variable
EC_e_			
-2Log-likelihood	230.70	240.56	EM_h_
Log-likelihood ratio	0.37	8.52	
CAO			
-2Log-likelihood	243.19	251.34	EM_ave_h_+EM_ave_v_
Log-likelihood ratio	1.41	10.27	

Compared with the full model which used all the ancillary data, a reduced model having a log-likelihood ratio greater than 1 was preferred according to Kerry and Oliver [44]. With regard to the reduced models for predicting EC_e_, dropping EM_v_ resulted in a log-likelihood ratio of 0.37, whereas removing EM_h_ resulted in a log-likelihood ratio of 8.52. This indicated that the removal of EM_h_ caused the greatest loss in predictive capacity for EC_e_, whereas EM_v_ had no effect on prediction performance given that the log-likelihood ratio was less than 1. This was also proven from Table 4 where EM_h_ had the most significant correlation with EC_e_. Therefore, EM_h_ was selected as the ancillary variable of EC_e_ and used to develop a linear regression and REML. When considering the reduced models for predicting CAO, dropping EM_ave_h_ and EM_ave_v_ resulted in log-likelihood ratio of 10.27 and 1.41, respectively, indicating that removing either EM_ave_h_ or EM_ave_v_ would produce loss in prediction capacity of CAO. As a result, both ancillary variables of EM_ave_h_ and EM_ave_v_ were retained and used to develop a multiple linear regression model and REML to predict CAO.

### Spatial distribution of ancillary variables

**Fig 2:**
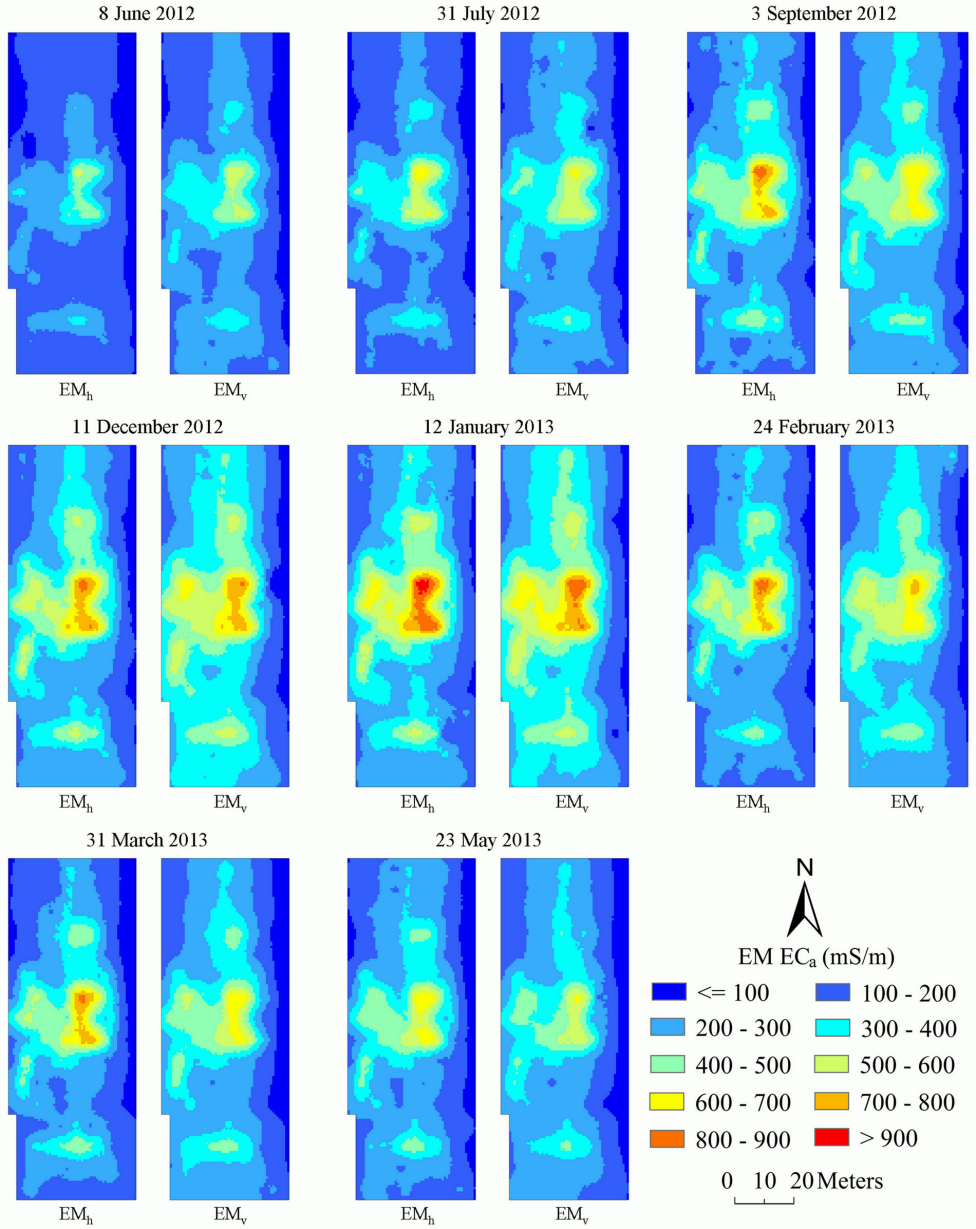
Spatial distribution of ancillary variables EM_h_ and EM_v_ on different survey dates.

Fig 2 shows the distribution maps of EMI measurements (252 locations) collected on eight survey dates. At the same survey period, EM_h_ and EM_v_ exhibited similar spatial patterns and this was not unexpected as the EM_h_ and EM_v_ were significantly correlated (Table 1). Also, spatial similarity was observed on different survey dates for both EM_h_ and EM_v_, and this could be ascribed to the uniform field management practices used in the field. Evident spatial trend was observed at various times. Taking the EM_h_ as an example, low EM_h_ measurements (< 100 mS/m) defined the areas at the eastern boundary of the field. Small-to-moderate (100–200 mS/m) and moderate-to large (200–400 mS/m) EM_h_ measurements, accounting for over 70% of the total area, mainly occurred in the north-central and south-central locations of the field. Large EM_h_ measurements (400–600 mS/m) characterized the central of the field and the extremely large EM_h_ measurements (> 600 mS/m) concentrated in patches within a relatively small region which used to be lower-lying area. High soil salinity resulting from poor drainage and water logging was a major cause of large EM_h_ measurements at these areas. This phenomenon was also observed in EM_v_ measurements.

### Digital mapping of soil EC_e_ and CAO

**Fig 3:**
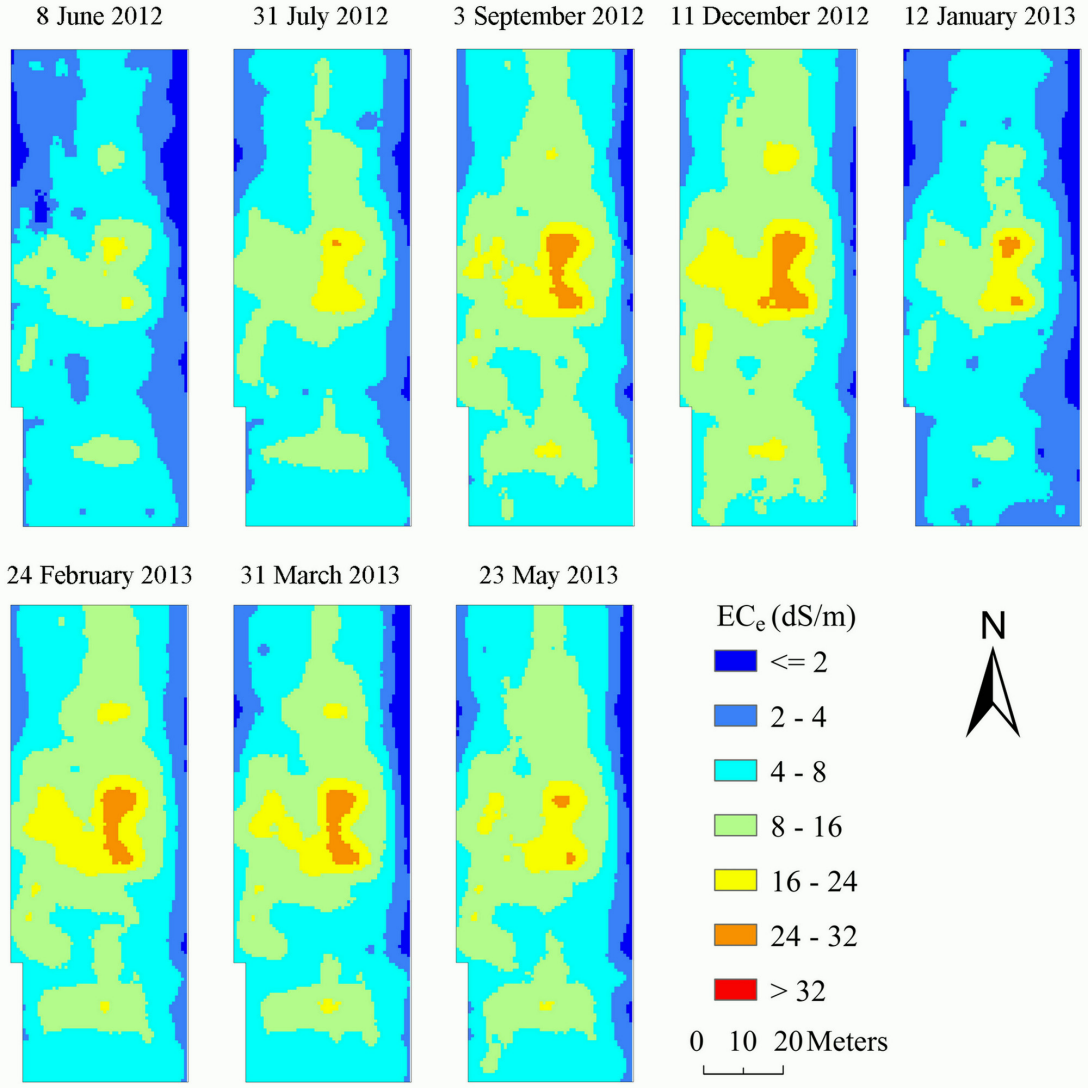
Spatial distribution maps of predicted soil EC_e_ (dS/m) on different survey dates.

Using the developed MLR model and REML, the spatial distribution maps of EC_e_ were generated from those of ancillary variables on different survey dates. The obtained distribution maps of EC_e_ are presented in Fig 3 and show agreement with those of EMI measurements at the corresponding time (Fig 2). From Fig 3, like a narrow band, the non-saline soil (EC_e_ < = 2 dS/m) and slightly saline soil (2–4 dS/m) mostly occurred at the east and north-west boundary of the field. Apparently, moderately saline soil (4–8 dS/m) and very saline soil (8–16 dS/m), which accounted for more than 75% of the field, were predominant from 8 January 2012 to 23 May 2013. Surrounded by very saline soil, extremely saline soil (> 16 dS/m) mainly concentrated at the central locations of the field on all the survey dates. In fact, the field investigation revealed that the presence of extremely saline soil was mainly attributable to the soil landscape which was located within a depression here.

**Fig 4:**
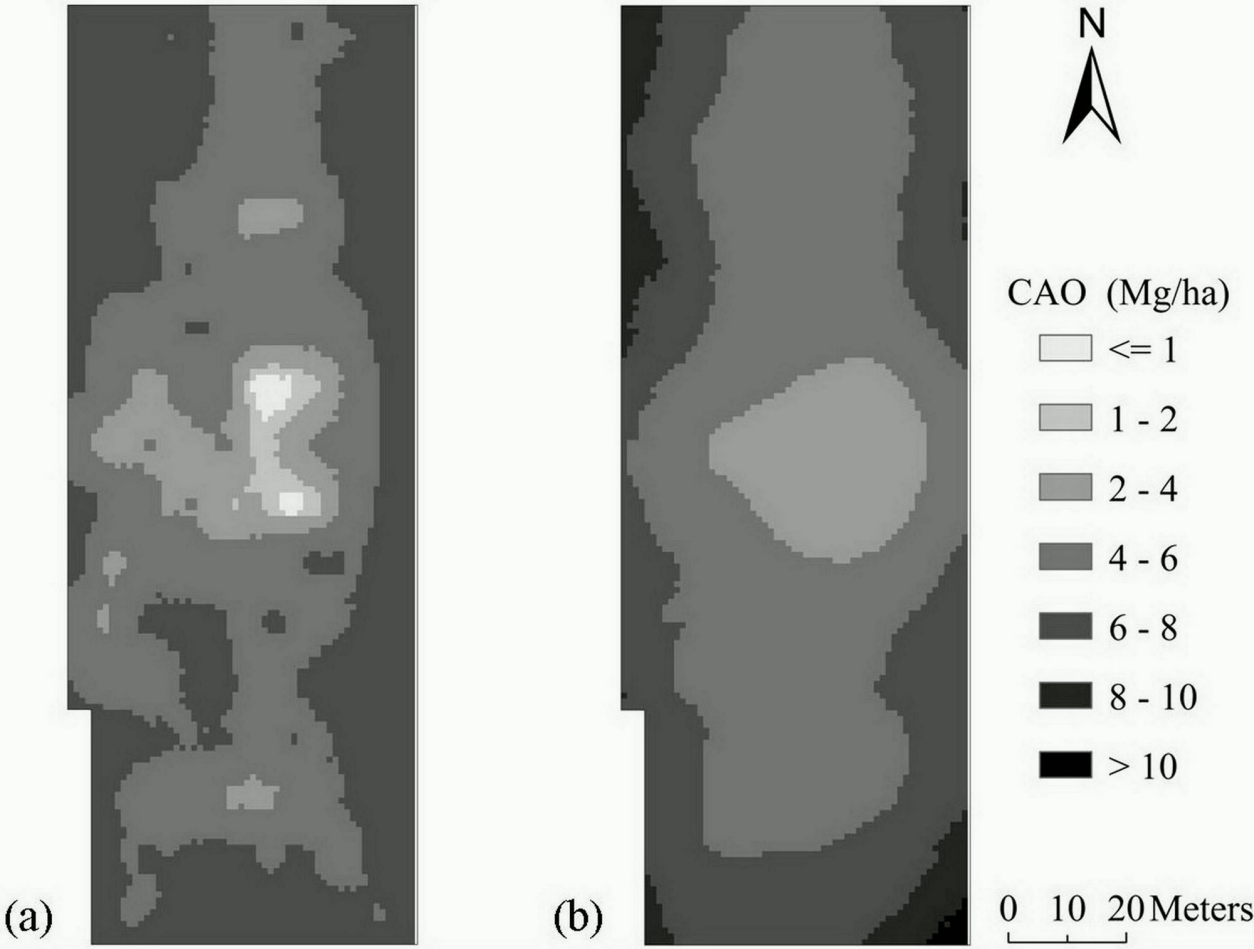
Spatial distribution maps of predicted CAO using a) fitted MLR and REML; b) raw crop sampling data.

Fig 4a exhibits the distribution map of CAO calculated from the fitted MLR model, REML and the average values of ancillary variables on the eight survey dates. As also shown in Fig 4b is the distribution map of CAO estimated from the raw crop date at 64 sampling locations. Obvious spatial similarity was observed between the two graphs, indicating that the spatial CAO was predicted from the ancillary variables with high reliability. In addition, compared with the graph generated using raw crop sampling data (Fig 4b), more details of the short range variation of CAO was reflected in the graph developed using REML and ancillary variables (Fig 4a). Generally, the spatial trend of CAO was opposite to that of EC_e_, indicating that high CAO mostly occurred at locations with low EC_e_, and vice versa. This showed agreement with Li et al. [45] and Yao et al. [2] who found that soil salinity was negatively associated with crop yield and the spatial pattern of soil salinity had strong impact on shaping that of crop yield in coastal region. On the other hand, this result was not occasional as the spatial distribution of both EC_e_ and CAO was developed from the same ancillary variables.

### Prediction precision assessment

**Fig 5:**
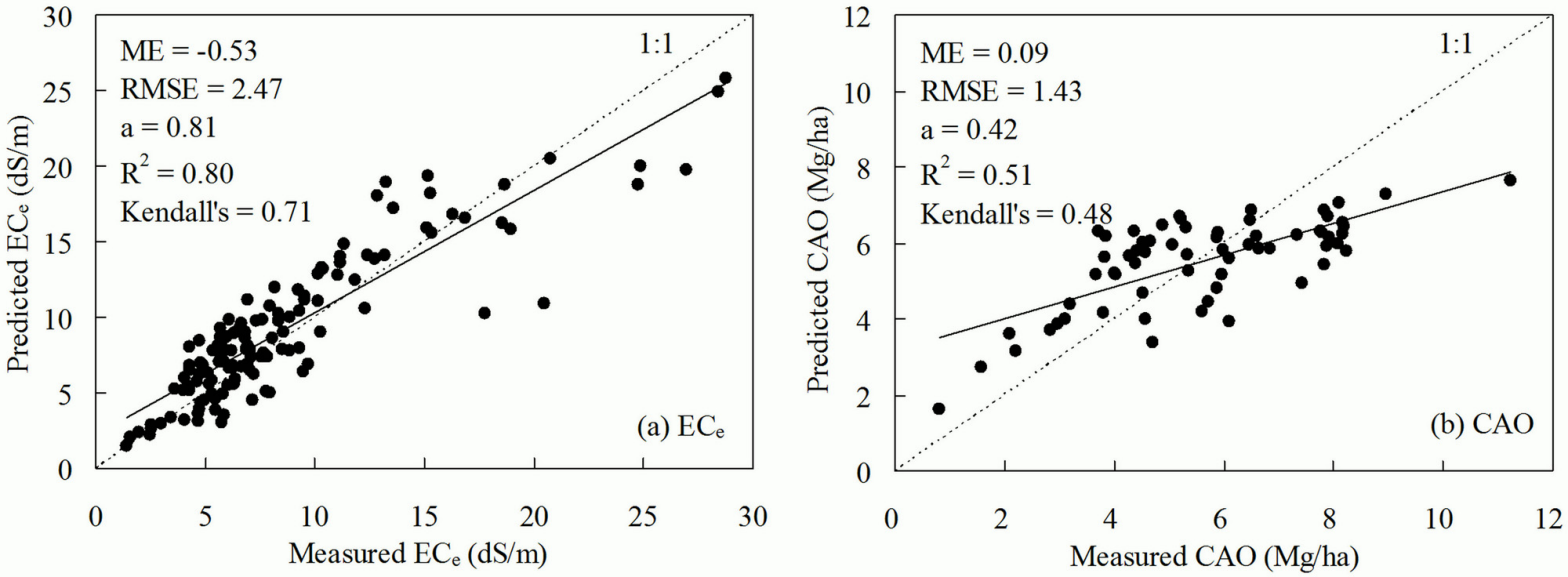
Prediction bias and accuracy of a) EC_e_ and b) CAO. Where ME is mean error, RMSE is root mean-square-error, a is the slope of the fitted regression line between measured and predicted values, R^2^ is coefficient of determination, Kendall’s is Kendall’s Tau-b coefficient of concordance.

Using the jackknifing procedure proposed by Huang et al. [46], the prediction performance was evaluated on the bias and precision between measured and predicted values. Fig 5 presents the results of predicted against measured EC_e_ and CAO values, plus the fitted regression line and prediction error statistics. Strong correlation was observed between the measured and predicted EC_e_ values with coefficient of determination of 0.80 and slope of 0.81. With regard to mean error (ME), an overestimation was observed for EC_e_, and this was indicated by most of points being above the 1:1 line. CAO prediction had a coefficient of determination of 0.51 and a regression slope of 0.42, it also got a ME of 0.09 and RMSE of 1.43, indicating that CAO prediction was less biased, however less precise, than EC_e_ prediction. A better measure of similarity between estimated and measured data was provided by Kendall’s Tau-b coefficient of concordance. This coefficient was 0.71 and 0.48 for EC_e_ and CAO, respectively, classified as moderate and significant at *p* < 0.01. These statistics confirmed the accuracy and reliability of spatial prediction of EC_e_ and CAO.

### Spatial and temporal dynamics of EC_e_

Table 7 presents the summary statistics of EC_e_ and percentage of soil salinity classes on the eight survey dates. The average EC_e_ ranged from 5.41 dS/m to 11.11 dS/m. A general increasing trend of EC_e_ was observed from 8 June 2012 to 12 January 2013 and then a decrease occurred from 12 January 2013 to 23 May 2013 across the field. The average EC_e_ on 8 June 2012 and 31 July 2012 was classified as moderately saline soil type (4–8 dS/m), whereas that on other survey dates pertained to very saline soil type (8–16 dS/m). With regard to the percentage of soil EC_e_ categories, area of non-saline and slightly saline soils was comparatively small, which accounted for 0.0%-6.57% and 6.09%-26.09% of the total field on eight survey dates, respectively. The proportion of extremely saline soil type ranged between 0.72% and 17.98%. Moderately saline (which ranged from 25.08 to 52.55%) and very saline soils (which varied from 14.06 to 50.85%) were predominant on all survey dates, accounting for 66.61%-81.44% of the total field.

**Table 7:** Summary statistics of EC_e_ in dS/m and percentage of soil ECe classes on various survey dates.

						Percentage of soil salinity classes in terms of EC_e_				
Survey date	Min.	Max.	Mean	St. D.	C_v_ (%)	< = 2.0 dS/m	2–4 dS/m	4–8 dS/m	8–16 dS/m	> 16 dS/m
8 June 2012	0.22	19.98	5.41	2.83	52.38	6.57	26.09	52.55	14.06	0.72
31 July 2012	1.29	24.54	7.04	3.74	53.03	2.35	15.81	51.28	26.98	3.58
3 September 2012	0.91	31.14	9.26	4.86	52.49	2.51	7.03	38.37	43.07	9.02
11 December 2012	1.63	30.68	10.14	5.25	51.75	0.74	8.87	28.48	48.46	13.45
12 January 2013	2.12	34.30	11.11	5.85	52.63	-	6.09	25.08	50.85	17.98
24 February 2013	1.73	30.78	9.23	5.01	54.31	0.32	10.06	39.80	39.05	10.77
31 March 2013	0.77	29.81	8.55	5.03	58.86	4.61	9.28	42.75	34.73	8.63
23 May 2013	0.93	25.17	8.45	4.38	51.90	3.92	10.14	39.47	40.50	5.97

**Fig 6:**
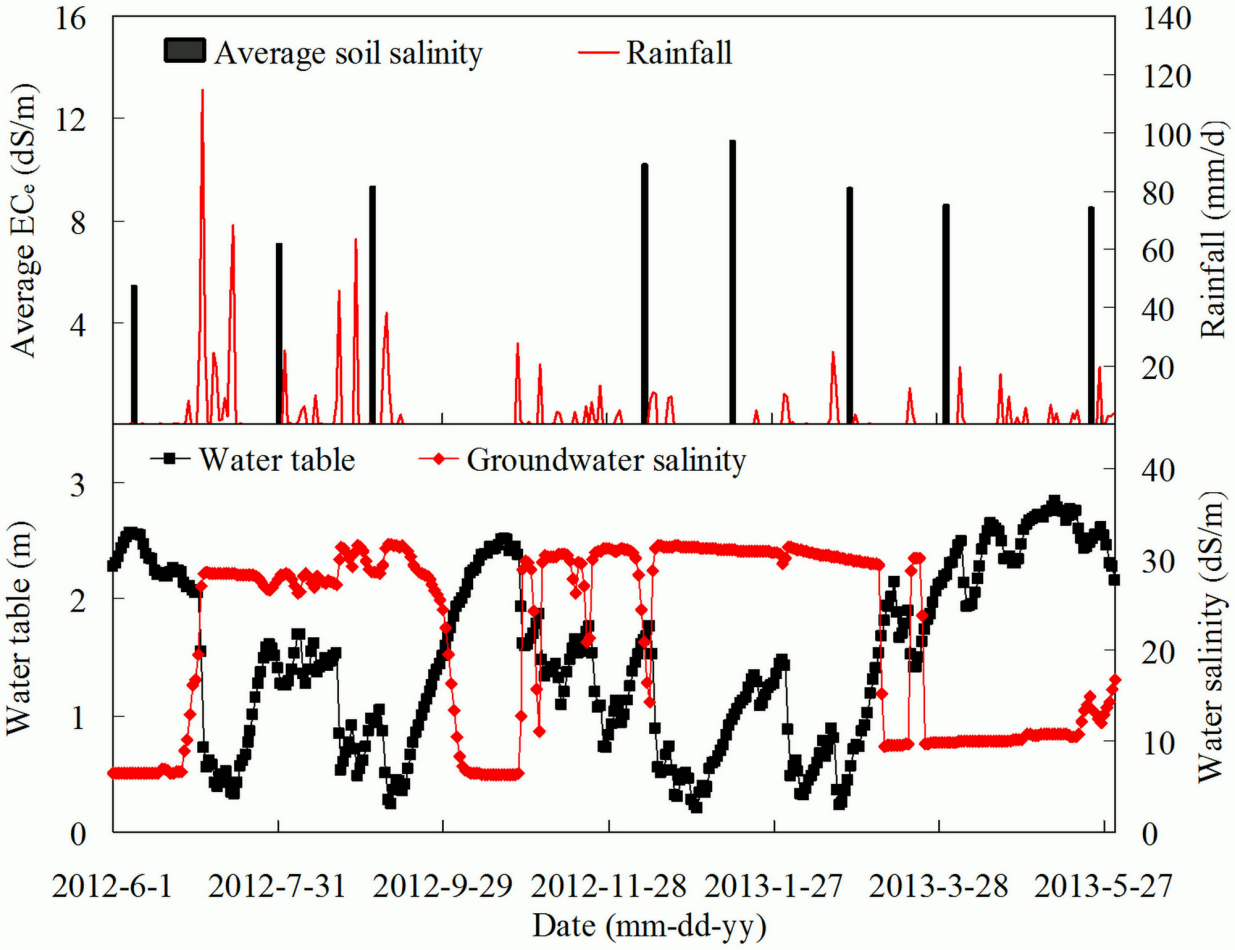
Temporal dynamics of average root zone EC_e_, rainfall, water table and groundwater salinity during the survey period.

The temporal dynamics of EC_e_ was further validated using the daily rainfall, water table and groundwater data shown in Fig 6. Apparently, water table well responded to the rainfall, indicating large amount rainfall resulted in the subsequent rise of groundwater and less rainfall generally led to the decline of groundwater. Taking water table as an example, it decreased from 2.05 m on 1 July to 0.32 m on 15 July with a total of 338.8 mm rainfall occurring during this period. Also, groundwater salinity varied with the rainfall and the fluctuation of water table, and high groundwater salinity was generally accompanied with shallow water table and vice versa. When considering the relationship between root zone EC_e_ and the meteorological and groundwater data, the date when high root zone EC_e_ occurred was characterized by shallow water table and high groundwater salinity. For instance, in response to water table of 2.57 m and groundwater salinity of 6.47 dS/m, the average root zone EC_e_ was 5.41 dS/m on 8 June. 2012. However, on 12 January 2013, the water table was 0.97 m and groundwater salinity was 31.09 dS/m with an average root zone EC_e_ of 11.11 dS/m. In fact, significant negative correlation was observed between root zone EC_e_ and water table and significant positive correlation was also observed between root zone EC_e_ and groundwater salinity (S2 File). This result indicated that the temporal dynamic of the average root zone EC_e_ of the experimental site coincided with the meteorological and groundwater data. Another indication was that shallow water table and high groundwater salinity had adverse impact on root zone salinity.

### Precision issues in CAO mapping

In this study, crop yield was predicted successfully using the apparent electrical conductivity measured by electromagnetic induction as ancillary variables. This was achieved based upon the correlation between EMI measurements and soil salinity as well as the correlation between soil salinity and crop productivity. Therefore, the uncertainty of spatial CAO mapping relied on not only the regression model between EMI measurements and crop yield but also the prediction accuracy of spatial distribution of EMI measurements. With respect to the regression model, a linear mixed-effects model, in which random variables were introduced for variance decomposition, was used to describe the relationship between EMI data and CAO. In many other studies, boundary line analysis, which was originally proposed to investigate fields where yield components could not reach their optimal values and to identify the most important limiting factors [47], was also used to determine the relationship between soil salinity and crop yield [48]. Boundary line analysis was not employed in this study as soil salinity measured by EMI measurements was proven to be the most important limiting factor of crop productivity in the coastal salt-affected farmland [2, 49]. When the reliability of spatial distribution of EMI measurements was considered, the prediction accuracy of ordinary kriging (OK) was satisfactory with ME of -8.45 dS/m and RMSE of 49.65 dS/m for EM_ave_h_, and with ME of -2.69 dS/m and RMSE of 33.82 dS/m for EM_ave_v_, and the corresponding determination coefficient (R^2^) of the regression between measured and predicted EMI measurements was 0.87 and 0.93, respectively. Furthermore, regression kriging (RK) method, which performed the estimation by adding the krigged residuals to the regression predictions was also not employed in this study, although RK method was reported to prevail over OK method in prediction accuracy when the ancillary variables were available [50]. The reason was that OK method had an advantage over RK method in crop yield prediction when only repeated EMI surveys were conducted.

Crop yield has high variability across fields and years as a result of complex interactions among different factors, including topography, soil nutrients, weather conditions, management practices and especially soil salinity in the coastal area [51]. The data of EMI measurements and crop yield, based on which the spatial distribution was investigated using digital soil mapping (DSM), was collected in just one year. Therefore, further efforts are needed to perform long-term EMI surveys and soil and crop data collection, to validate whether our findings would be also useful over time and in different salt-affected regions, management systems, metrological conditions and land use patterns.

## Conclusions

Repeated electromagnetic induction (EMI) surveys were performed across a salt-affected farmland in coastal regions of Jiangsu Province, China during the study period. Significant correlation between apparent electrical conductivity (EC_a_) and soil EC_e_ (electrical conductivity of saturated paste extract) and crop yield allowed for rapid characterization of the spatio-temporal variation in soil salinity and crop annual output (CAO) using EC_a_ survey data. Results of linear mixed-effects model and log-likelihood analysis showed that EM_h_ could be used as a solo predictor for EC_e_ calibration, whereas both EM_h_ and EM_v_ should be used to meet the need of CAO calibration. Spatial patterns of soil salinity and CAO, as derived from EMI survey data, showed agreement with those generated from raw data with low bias and high reliability. Spatial soil salinity exhibited temporal dynamics with the increasing trend from 8 June 2012 to 12 January 2013 and decreasing trend from 12 January 2013 to 23 May 2013, which coincided with the meteorological and groundwater conditions during those periods. Spatial distribution of CAO showed that crop yield could be predicted using ordinary kriging with satisfactory accuracy.

It is concluded that the cost-effective and efficient EMI surveys, as one part of multi-source data for digital soil mapping, can be successfully used to characterize the spatial and temporal variability of soil salinity and to estimate potential crop yield spatially. The methodology of this study can be used as guidance for researchers who are interested in understanding soil salinity development as well as land managers aiming for appropriate soil salinity management strategies and maximum efficiency of crop outputs. In order to characterize the spatio-temporal variations in soil salinity and crop yield on larger scales, more sophisticated EMI instruments (e.g. DUALEM-421) as well as remote sensing data (e.g. MODIS satellite imagery) can be integrated for digital soil mapping [4, 52].

## Supporting Information

S1 File EMI survey data on the 8 survey dates were collected with EM38 in the horizontal (EM_h_) and vertical (EM_v_) dipoles, respectively. 128 calibration sites were sampled on the two survey dates and rootzone electrical conductivity of saturated paste extracts (EC_e_) were measured for the calibration of EMI measurements. Crop annual output (CAO) was determined by summing corn yield and barley yield during the survey period.
(XLSX)

S2 File Daily average water table and groundwater salinity was obtained from the hourly collected data of CTD-Divers. Daily rainfall from June 2012 to May 2013 was obtained from the hourly collected data of the weather station.
(XLSX)
